# Implementation and evaluation of an algorithm for the management of scabies outbreaks

**DOI:** 10.1186/s12879-019-3818-5

**Published:** 2019-02-28

**Authors:** Simon M. Mueller, Stefan Gysin, Michael Schweitzer, Simon Schwegler, Peter Haeusermann, Peter Itin, Thomas Bart, Ruth Spieler Denz, Thomas Steffen, Richard Kuehl, Andreas F. Widmer, Oliver Brandt

**Affiliations:** 1 Department of Dermatology, University Hospital Basel, Petersgraben 4, 4056 Basel, Switzerland; 2 Department of Health, Medical Services, Social Medicine, 4056 Basel, Canton of Basel-City Switzerland; 3 Department of Infectious Diseases and Hospital Epidemiology, University Hospital Basel, 4056 Basel, Switzerland

**Keywords:** Scabies infestation, Epidemic outbreak, Infection control, Permethrin, Ivermectin

## Abstract

**Background:**

Infestations with scabies mites are a global burden affecting individuals of all ages, classes and ethnicities. As poor sanitation and overcrowding favor the transmission of this highly contagious disease, epidemic outbreaks are frequently observed among displaced persons and asylum seekers. Due to the growing influx of refugees during the last years, public health authorities in host countries are frequently confronted with the challenge to treat individuals with diagnosed or suspected scabies promptly and effectively to avoid further spreading of the infestation.

This study aimed to establish a straightforward and efficient algorithm for rapid screening and treatment of large numbers of patients with confirmed or suspected scabies infestations.

**Methods:**

Forty-eight individuals (58% males, mean age 22.4 yrs.) from Syria with suspected scabies infestation were allocated to 3 colour-coded groups: (1) no signs or symptoms of infestation, (2) itch only, and (3) itch and typical skin lesions. Patients were treated with a single (group 1) or two doses of oral ivermectin at an interval of 7 days (group 2), or with a combination of 2 doses of ivermectin plus 2 applications of permethrin ointment at an interval of 7 days (group 3). Follow-ups were performed 4 weeks after initial treatments.

**Results:**

All individuals with signs and/or symptoms of infestation had improved skin lesion; in 10/11 (90.9%) lesion had completely resolved. All individuals with initial itch only (*n* = 32) reported improvement of its intensity or complete resolution. None of the patients of group 1 developed itch or skin lesions. The algorithm was reapplied in 4 individuals (8.3%) after 4 weeks and the outbreak was completely controlled after 8 weeks. Colour-coding ensured fast flow of information between health-care providers at the interfaces of the algorithm.

**Conclusions:**

Our algorithm proved to be both highly efficient for treatment of large numbers of patients with suspected or diagnosed scabies infestation as well as for prevention of spreading of the disease. Hence, this algorithm is well suited for the management of scabies mass outbreaks.

## Background

Infestation with *Sarcoptes scabiei var. hominis* mites is a global burden affecting individuals of all ages, classes and ethnicities [1–4]. According to Vos et al. more than 200 million people worldwide suffer from this ectoparasitic infection, which is endemic in many tropical regions, especially in low-income communities [5]. Signs and symptoms of scabies are the result of a hypersensitivity reaction to proteins released by the mites and usually manifest with severe itch and characteristic skin lesions consisting of burrows and erythematous papules that are often excoriated. Typical lesions are most commonly found at the webs of the fingers, the flexor aspect of the wrists, the torso - particularly on the genital region - and on the feet. As scratching compromises the barrier function of the skin, bacterial superinfections frequently occur, occasionally resulting in sepsis, acute poststreptococcal glomerulonephritis and acute rheumatic fever [4, 6–8]. Overcrowding and poor sanitation favour the transmission of this highly contagious disease and epidemic outbreaks are thus regularly observed among displaced persons [2, 9]. Due to the continuing arrival of refugees in Europe, public health authorities in host countries are frequently confronted with the challenge of treating individuals with suspected or diagnosed scabies promptly and effectively to avoid further spreading of the infection [9–12]. According to a recent retrospective cohort study from the Netherlands, 47.4% of asylum seekers from Eritrea and Ethiopia had clinical signs of scabies [9] and another recently published study analyzing health issues in asylum seekers in Germany found that routinely physical examination and treatment of scabies was justified in addition to tuberculosis screening [13]. However, to our knowledge, the Netherlands were the only European country that implemented a scabies intervention program at the beginning of the so-called refugee and migrant crisis [9].

Today, permethrin ointment and oral ivermectin are the cornerstones of scabies treatment as both drugs are highly effective, very well tolerated and relatively inexpensive [3, 14, 15]. The “European guideline for the management of scabies infestation” recommends to treat patients either with two applications of permethrin 5% cream or oral ivermectin 200 μg/ kg body weight each one week apart [3]. However, although the response rate to the two drugs is considered high, we have seen several patients in the past, particularly refugees, who were not cured despite the application of this regime. Possible reasons for this observation could be inadequate application of permethrin and insufficiently followed general measures (e.g. decontamination of clothes, avoidance of close contacts).

To date, only vague recommendations for the treatment of mass outbreaks are available [3, 14, 15]. While the European guideline recommends the use of two doses of ivermectin one week apart and does not explicitly consider the application of permethrin as a therapeutic option [3], the Centers of Disease Control and Prevention (CDC) guideline not even suggests any drugs to combat scabies mass infestations and merely mentions that it can be difficult to control institutional outbreaks [14, 15]. Furthermore, none of the two guidelines deals with organizational issues - such as tracing the source of infection, triage and on-site treatment, instruction of patients not familiar with the language spoken, organization of follow-up visits etc. - which health workers are frequently faced with in the management of mass outbreaks.

To resolve this shortcoming, we developed an algorithm that enables the management of large numbers of individuals with suspected scabies despite language barriers. We evaluated its practicality on 48 refugees from Syria who were referred to our clinic because of suspected scabies infestation.

## Methods

### Study design

In April 2017, the Department of Health, Medicine and Social Medicine of the Canton of Basel-Stadt, Switzerland, sent a group of 48 Syrians to our dermatology department in a single afternoon to assess and, if necessary, treat suspected scabies. The majority of the patients were residing in local refugee hostels with confined living conditions and were identified by the local health authority as potential carriers of infection.

This algorithm was developed to screen and treat as many patients as possible with suspected scabies in a short time despite language barriers and to inform them about necessary behavioural measures, to heal them from the disease and prevent its spreading. The scabies intervention team assembled to accomplish this task, consisted of dermatologists, infectious disease specialists, nurses/nurse aids, members of the patient administration service and translators. This interdisciplinary team was experienced in the management of a previous scabies outbreak in an intensive care unit affecting patients and healthcare workers [16]

### Setting

Dermatology out-patient clinic of a university teaching and tertiary care center.

### Inclusion/ exclusion criteria

Subjects living in the same household and close relatives/friends of the them were classified as at risk of being affected by scabies according to the above mentioned guidelines. Individuals who, according to the local health authority, did not live in the same household, had no close contact with scabies patients (no hugs or repeated skin contact, no sharing of towels, clothes, bed etc.) were excluded from participation in the study and were not examined in our department.

#### Infection control intervention

**Fig. 1:**
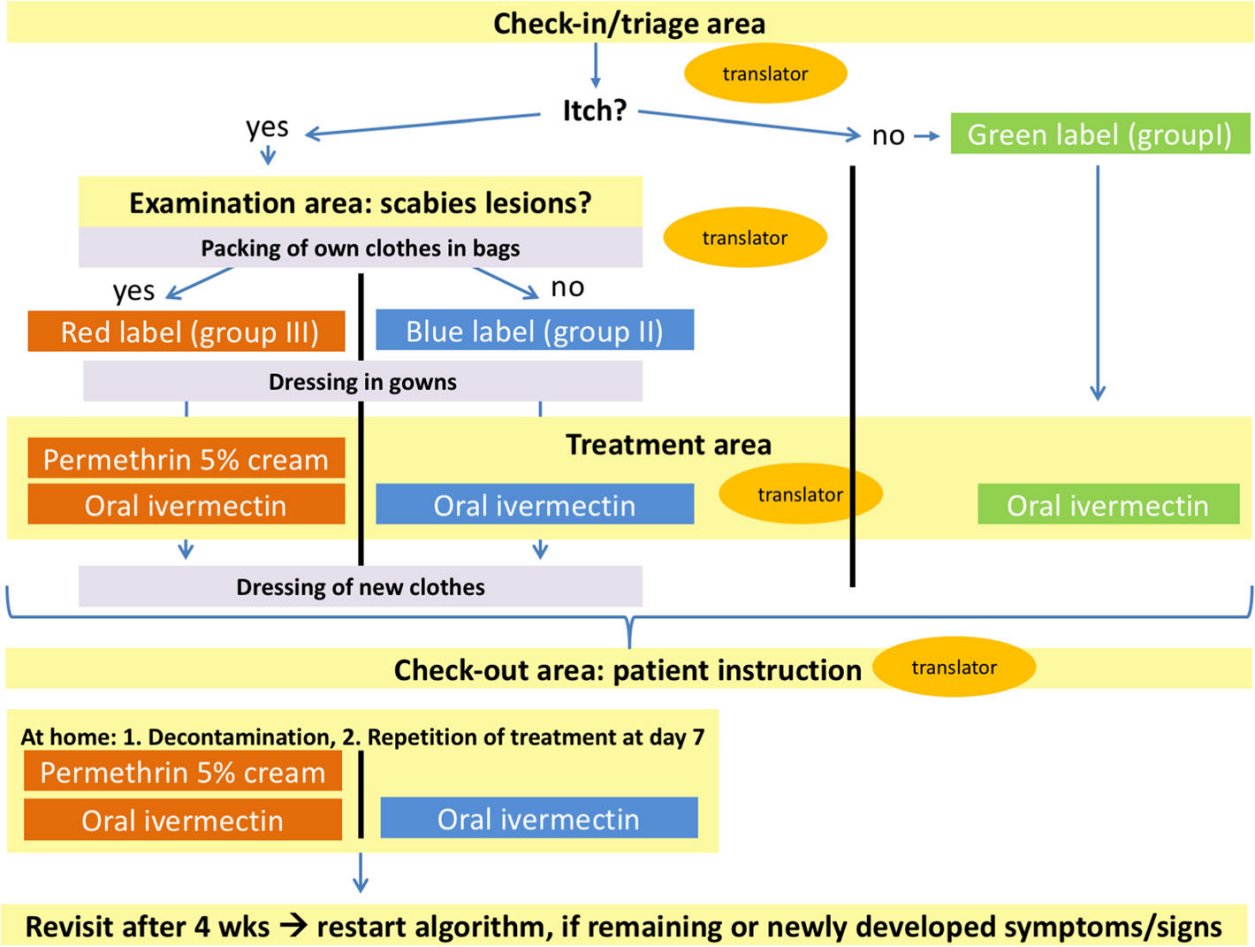
Algorithm used for the treatment of patients with suspected and confirmed scabies infestations. Four separate areas (check-in/ triage area, examination area, treatment area, and check-out area) involving different health-care providers were used at the different steps of the algorithm. Patients with neither symptoms nor signs were assigned a green label, those with itch only a blue label and patients with itch and skin lesion typical for scabies infestation a red label

To manage the task of treating large numbers of patients within a short time, we developed and employed an algorithm for the management of ectoparasite outbreaks (Fig. 1). The algorithm differs from the European- and CDC guidelines [3, 14, 15] in that we apply ivermectin in a dose depending on the presence of signs and/or symptoms and chose a combined therapy (permethrin 5% and ivermectin 200 μg/kg body weight) for patients with clinically confirmed scabies based on physical examination. This adjustment was made based on our experience that some refugees remained infested despite treatment according to the European guideline.

We proceeded as follows: After informing the individuals about the subsequent procedure, focused histories with special considerations of the type and duration of itching, assessments of allergies, chronic skin diseases, comorbidities and current living conditions were taken. Thereafter, if necessary, they were examined by dermatologists following the procedures and recommendations previously described by other authors [9, 17, 18]. In brief, individuals were dressed in temporary clothes and investigated for the presence of inflammatory and red-brown papules/nodules and burrows at the characteristic predilection sites of scabies (e.g. interdigital spaces, wrists and ankles, axillae, areolar regions, waist, groin and genitals). Dermoscopy was performed to assess skin lesions, especially if they had only existed for a short time. The delta wing sign was regarded as proving the presence of a Scabies mite. In order to protect the privacy of the patients, the physical examination was carried out by a same-sex dermatologist in a room that was not visible from the outside, as was any subsequent topical treatment by a nurse that might be necessary.

According to their signs and symptoms, patients were subsequently allocated to one of a total of three groups and each with a specific colour assigned. Individuals with neither itch nor signs were allocated to group I (green), individuals with itch but no signs of scabies to group II (blue) and individuals with both itch and signs were allocated to group III (red). We applied adhesive coloured stickers to visible areas such as the neck or forearms of the individuals and also marked their medical records accordingly. Following group assignment, patients were treated with either a single dose of oral ivermectin 200 μg/kg (group I), 2 doses of oral ivermectin 7 days apart (group II) or a combination of 2 doses of oral ivermectin plus 2 applications of permethrin 5% ointment 7 days apart (group III). Dosages of ivermectin were adapted in children and potentially pregnant patients according to the manufacturer’s recommendations. Application of the medications 7 days after initial treatment was done by the patients themselves after detailed medical instruction at visit 1. Group II and III patients were reexamined after 4 weeks and in case of persistent signs and symptoms and retreated as described above. A follow-up examination of these individuals was then carried out 4 weeks later (visit 3). Patients of group I were contacted by telephone 4 weeks after their first visit and asked about any complaints that may have occurred in the meantime. All patients were urged to contact us immediately in case of new or recurrent symptoms during the whole study period of 8 weeks and also thereafter.

Immediately after the treatment, patients of group II and III put on new or freshly washed clothes brought by them and stowed the potentially contaminated garments in closable plastic bags handed over by us. Written instructions on how to behave, decontaminate textiles and apply medications were given to the patients [3, 19]. Of note, at each of the different stages of this algorithm, a translator was present (see Fig. 1). During the procedure, all health care professionals and translators involved wore nitrile gloves and fabric dresses to protect themselves from transmission of scabies mites.

## Results

Twenty-eight of the forty-eight (58%) patients were males. Mean age was 22.4 yrs. (range 16 months to 68 years), 20 individuals were between 16 months and 18 yrs. of age. All but 8 lived in households with family structures consisting of 4–9 members. The tracing of contacts between the affected families revealed that one family was most likely the source of the spreading of the infestation. Sixteen out of the 48 individuals (33.3%) complained of recently appeared and persistent itch, one of which suffered from preexisting but currently well-controlled atopic eczema. Based on their signs and symptoms, 32 individuals (66.7%) were assigned to group I (green), 5/48 (10.4%) to group II (blue) and 11/48 (22.9%) to group III (red) at visit 1.

**Fig. 2:**
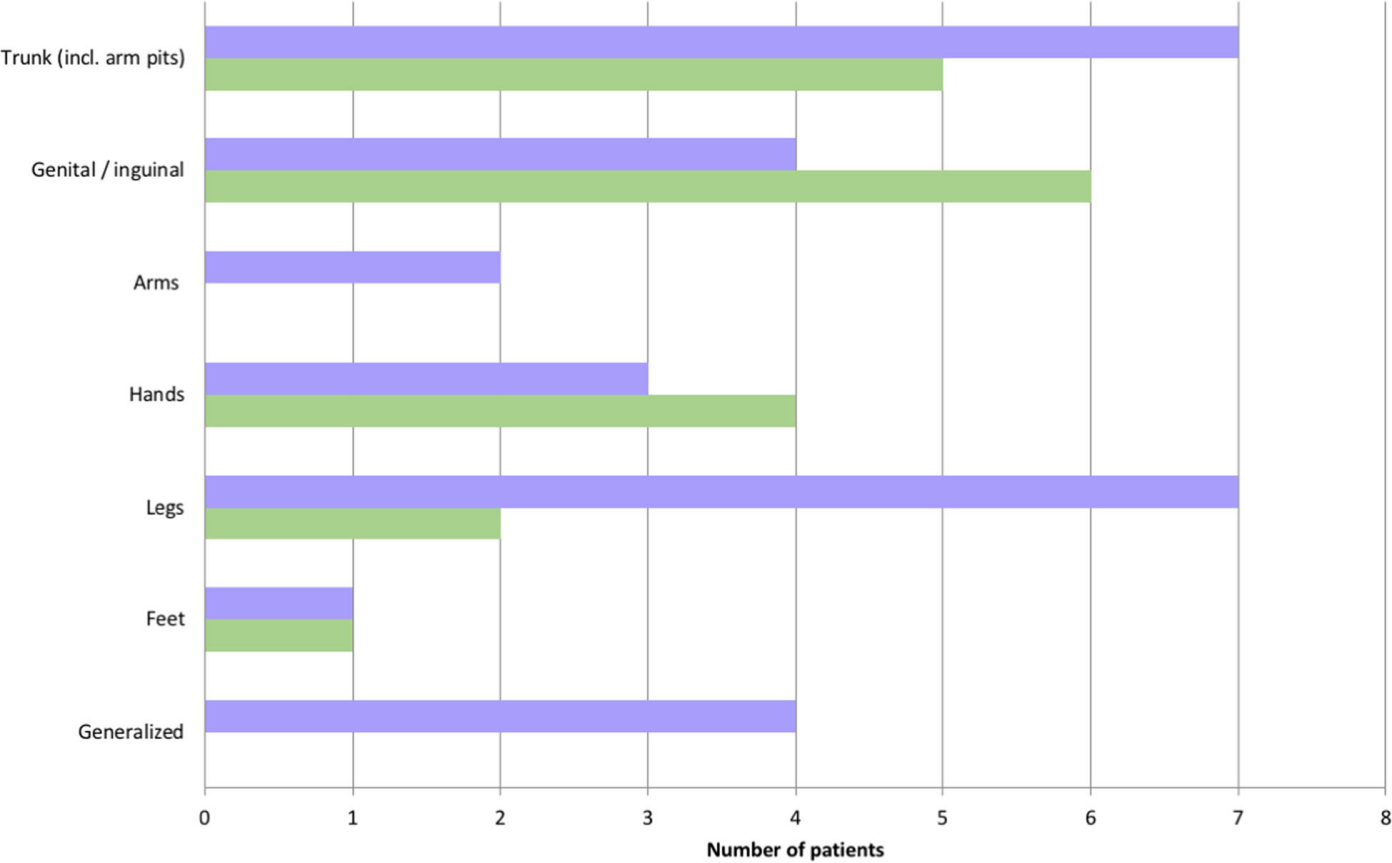
Localisation of itch (blue bars) and scabies lesions (green bars) in 16 patients

Analyses of the localization of itching and skin lesions revealed that they did not always match (Fig. 2). While patients reported that the trunk and legs were often affected by itching, scabies skin lesions were most frequently found in the genital region. Of note, none of the patients showed crusted lesions.

**Fig. 3:**
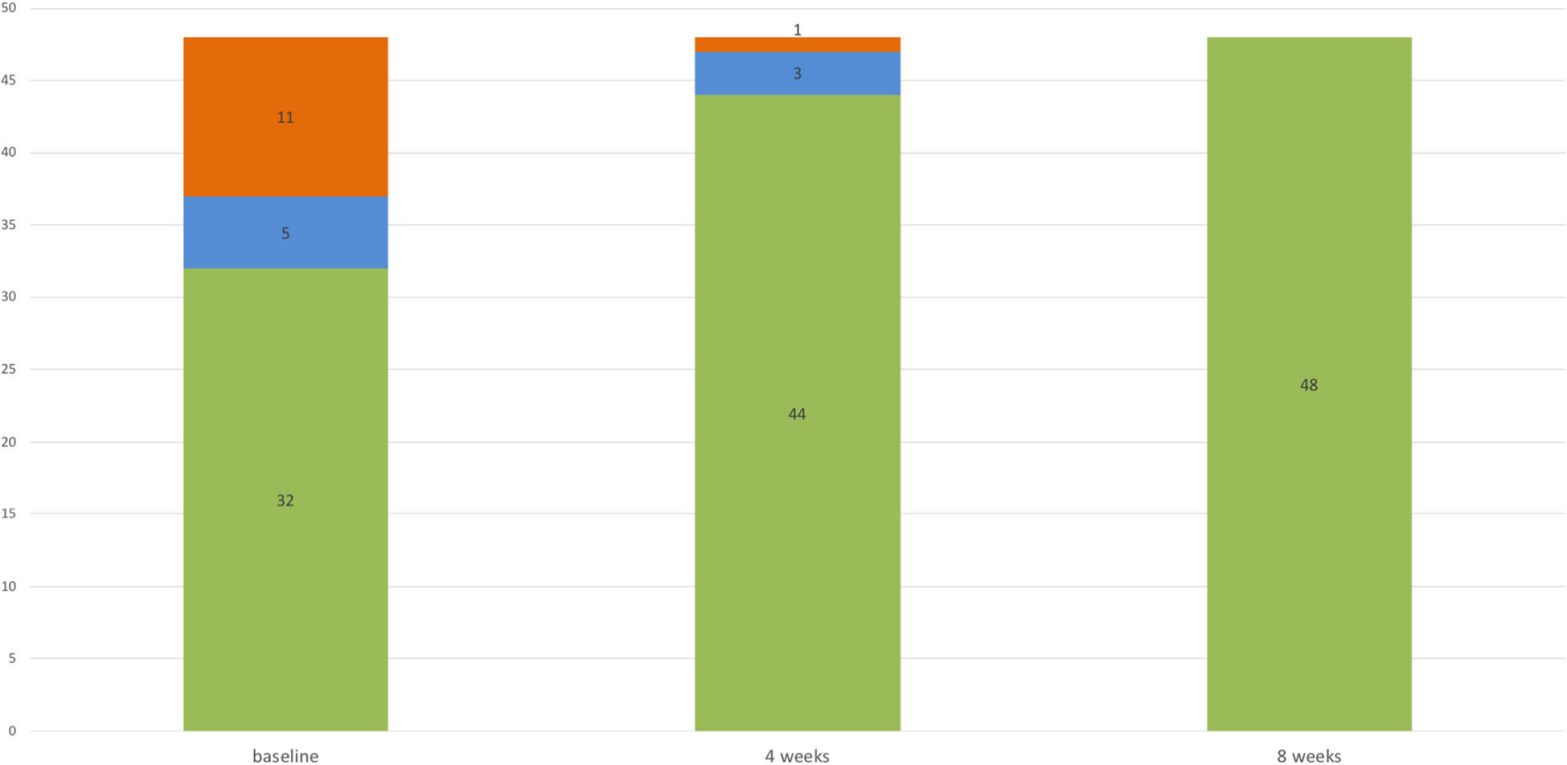
Distribution of patients (*n* = 48) according to their signs and symptoms at baseline (visit 1), after 4 (visit 2) and 8 weeks (visit 3). Individuals were assigned to the following groups and treated as described in the Methods section: neither itch nor signs (group I, green parts of columns), itch without skin lesions (group II, blue parts of columns), itch and skin lesions (group III, red parts of columns). Figures in columns represent numbers of patients of each group at the different time points

At visit 2 (4 weeks after the initial consultation), 11/16 (69%) symptomatic individuals (group II and III) reported an improvement of their symptoms. In all individuals with initial itch only (group II, *n* = 5), reported itch intensity had markedly improved and 2 patients were totally free of symptoms. None of the individuals in groups I (green) and II (*n* = 37) had developed scabies lesions and none of the individuals in group I (*n* = 32) complained about itch as evaluated by telephone interviews. All individuals, who initially exhibited signs of scabies (group III, *n* = 11) had improved, in 10/11 (90.9%) cases skin lesions had completely and in 1/11 partially resolved. The latter individual with potential persistent scabies and the subject with itch only (n = 3) were again treated according to our algorithm. Four weeks later at visit 3, these 4 patients were also free of symptoms (Fig. 3). No adverse side effects to permethrin or oral ivermectin were reported by the treated individuals or observed by us. Also of note, despite intensive contact with the scabies-infected patients, none of our health care professionals involved became infested.

## Discussion

Scabies is one of the leading health challenges in the current European migrant crisis for both public health authorities and health care institutions [9–11, 19–23]. Although the European guideline provides recommendations for the treatment of scabies mass outbreaks, it does not make any concrete proposals for organizing its practical implementation. In collaboration with the local health care authority, we developed and tested a colour-coded algorithm, which is based on clinical signs and symptoms, to manage large numbers of refugees and other individuals with suspected or confirmed scabies. By following this algorithm, we were able to triage, examine, treat and inform a large number of patients within a short time.

To our knowledge, there is currently no algorithm available that describes the management of scabies mass outbreaks. Publications on institutional (e.g. hospitals, residential care homes) [16, 24–27] or endemic scabies [17, 28] reported various strategies to combat outbreaks, but none provided a description for the management procedures in concrete terms. However, the key steps to control scabies outbreaks are basically the same, irrespective of the setting, and include (I) a meticulous planning of the entire procedure, taking into account special constellations (e.g. necessity of involving translators in case of language barriers), (II) registration of all persons at risk and their education about important behavioural measures (III) simultaneous examination and treatment of all persons at risk, (IV) decontamination measures for potential transmitters of infection, such as clothing and other textiles, cuddly toys, etc., and (V) follow-ups including retreatments if necessary [16, 25–27, 29].

We have implemented all five points in our algorithm. The colour-coded process allowed us to customize the scabies management: People without signs of infestation (Group I, green) were managed, treated and discharged within minutes, giving more time and human resources to Group II (blue) and especially Group III (red). The colour-coding ensured a fast and clear flow of information and thus a smooth process at the interfaces of our algorithm. Most time-consuming in our algorithm were steps involving translators (e.g. history taking, instructions regarding treatment, decontamination measures and follow-ups).

Whereas the main steps were similar in the aforementioned studies, larger variety was reported in terms of drug choice for prophylaxis (permethrin or ivermectin) and treatment (permethrin and/or ivermectin) and regarding the dosing regimens (once or twice) - partly because of inconsistent approvals of ivermectin in European countries and regional permethrin resistances [3, 16, 24–27, 29–31]. Prior to our standardized management of the outbreak, we had experienced that monotherapy twice with either permethrin or oral ivermectin was not effective in some patients and for whatever reason, especially in refugees. Therefore, we treated patients with scabies twice with permethrin and oral ivermectin, as recommended in the European guideline for crusted scabies [3]. Furthermore, we treated individuals with itch but without scabies lesions (group II, blue) with two doses of oral ivermectin. Unfortunately, the European guideline does not clearly address this constellation in mass population treatment [3]. It states that a single dose of oral ivermectin is effective and that a second dose is recommended (level of evidence Ib), although the importance of this additional dose needs to be evaluated in future studies [3]. When implementing these imprecise recommendations into clinical practice, there is the risk of undertreating symptomatic, and thus possibly affected patients [26], with only a single dose of oral ivermectin, whereas asymptomatic individuals may unnecessarily be treated with two doses. Our results support the notion of differentiating mass treatment in this particular point: asymptomatic individuals can be treated with a single dose of oral ivermectin, symptomatic individuals, who do not have cutaneous signs of scabies, with two doses of the drug at one week intervals. The two treatments with a combination of permethrin and oral ivermectin, as recommended by the European Directive [3], have proven successful in our setting.

In addition, the efficiency of our algorithm is demonstrated by the fact that we were able to control the outbreak within only 8 weeks, while a retrospective study that examined institutional outbreaks over a period of 30 years identified 3 months as the usual time needed [32].

In some of the patients of our cohort, scabies lesion were mainly localized in the genital region. Similar observations were described by Beeres et al., who investigated more than 1300 refugees with scabies from Eritrea and Ethiopia, who had recently arrived in the Netherlands [9]. The authors found that scabies lesions were most common in the genital region and on the hands, each with more than 30%. From these figures we conclude that inspection of these body regions is pivotal, especially in refugees from countries where scabies is endemic.

The individuals in this study were surprisingly compliant, all participants were reached by telephone at the agreed times and appeared at the scheduled follow-up examinations. One reason for this reliability might have been the detailed information about the disease and necessary therapeutic measures, which underlines the importance of professional translators for this procedure.

## Conclusions

Our algorithm is well suited for the management of scabies mass outbreaks, as it is easy to implement, enables the coordinated and rapid management of a large number of patients, has high treatment success rates and optimally involves those affected in the process.

## Abbreviations

CDC: Centers of Disease Control and Prevention
